# Platelets treated with pathogen reduction technology: current status and future direction

**DOI:** 10.12688/f1000research.20816.1

**Published:** 2020-01-23

**Authors:** Wen Lu, Mark Fung

**Affiliations:** 1 Section of Transfusion Medicine, Robert Tomsich Pathology & Laboratory Medicine Institute, Cleveland Clinic, Cleveland, OH, USA; 2 Department of Pathology and Laboratory Medicine, University of Vermont Medical Center, Burlington, VT, USA

**Keywords:** pathogen reduction technology, PRT-treated platelets, pathogen-reduced platelets, transfusion-transmitted infections, septic transfusion reactions, HLA alloimmunization

## Abstract

Allogeneic platelets collected for transfusion treated with pathogen reduction technology (PRT), which has been available in some countries for more than a decade, are now increasingly available in the United States (US). The implementation of PRT-treated platelets, also known as pathogen-reduced platelets (PRPs), has been spurred by the need to further decrease the risk of sepsis associated with bacterial contamination coupled with the potential of this technology to reduce the risk of infections due to already recognized, new, and emerging infectious agents. This article will review available PRP products, examine their benefits, highlight unresolved questions surrounding this technology, and summarize pivotal research studies that have compared transfusion outcomes (largely in adult patients) for PRPs with non-PRT-treated conventional platelets (CPs). In addition, studies describing the use of PRPs in pediatric patients and work done on the association between PRPs and HLA alloimmunization are discussed. As new data emerge, it is critical to re-evaluate the risks and benefits of existing PRPs and newer technologies and reassess the financial implications of adopting PRPs to guide our decision-making process for the implementation of transfusing PRPs.

## Background

Therapeutic and prophylactic platelet transfusions are lifesaving therapies. Similar to other medical interventions, the transfusion of platelets is not without risk. In contrast to other blood products, stored refrigerated or frozen, platelets are stored at room temperature, making them more likely to become contaminated with bacteria and cause septic transfusion reactions (STRs) ^1^. In the US, despite the mandatory requirement to test all platelet collections by performing primary bacterial culture on a sample obtained from the product, it is estimated that 1 in every 5,000 platelet collections is contaminated by bacteria and escapes detection ^2^, posing the risk of a STR. Various strategies have been proposed and implemented to further reduce the risk of bacterial contamination of platelets. In the US, pathogen reduction technology (PRT) is one of the options accepted by the FDA. PRT has already been in use in Europe for more than 15 years. Although pathogen-reduced platelets (PRPs) have a good record of efficacy and safety, a number of questions and concerns still remain. ****

This article will provide a summary of currently available PRP products and the key publications that laid the groundwork for their adoption. More recent work that details the areas of debate and concern regarding the use of platelets treated with PRT are reviewed. In the conclusion, critical questions that future research must address regarding PRPs are highlighted.

## Products available

PRT causes irreversible damage to the nucleic acids of bacteria, viruses, and parasites, preventing their replication and thereby decreasing transfusion-transmitted infections (TTIs). Three technologies are currently available for the treatment of platelets: INTERCEPT (Cerus Corp., Concord, CA, USA), Mirasol (Terumo BCT Inc., Lakewood, CO, USA), and THERAFLEX (MacoPharma, Mouveux, France). All three demonstrate clinically significant reduction of bacteria in platelets ^3, 4^. Recent reviews by Gravemann, Yonemura, and Schubert summarized data showing their effectiveness against bacteria and other infectious agents that are nucleic acid based ^5– 7^.

INTERCEPT uses a synthetic psoralen compound, amotosalen, and ultraviolet A (UVA) light to prevent nucleic acid replication, thereby inactivating infectious agents and lymphocytes ^8^. After INTERCEPT treatment, an adsorption step removes nearly all residual amotosalen ^9^. Countries in Europe were the earliest adopters of PRPs when INTERCEPT was given Conformité Européenne (CE) designation by the European Union (EU) in 2002 ^10^. Belgium adopted INTERCEPT PRPs by royal decree in 2009. In Switzerland, PRPs were introduced in 2011 and implemented nationwide within 12 months ^11^, followed by France in 2017. Based on national hemovigilance data from Europe, there have been no confirmed STRs after the transfusion of 227,797, 167,200, and 214,293 PRPs in Belgium, Switzerland, and France, respectively ^12^. An updated study from Switzerland reported no confirmed cases of transfusion-transmitted bacterial infection after the transfusion of 205,574 INTERCEPT PRPs ^11^. In 2014, the INTERCEPT system became the first PRT to be approved for use in the US by the FDA and is currently in a post-marketing surveillance study (PIPER, identifier: NCT02549222). In 2018, it was licensed in Canada.

Mirasol is another PRT that uses riboflavin (vitamin B2) as the light-activated photochemical and broad-spectrum UVA/UVB illumination. Riboflavin and its byproducts are naturally occurring and do not have any known side effects. Thus, there is no removal after treatment, as it is not believed to be necessary. Mirasol is approved for use in Europe (CE designated in the EU in 2007), Russia, and the Middle East and is currently being evaluated in the US in a phase III randomized clinical trial (MIPLATE, identifier: NCT02964325) ^13^.

A third PRT, THERAFLEX, uses UVC and vigorous shaking ^5^ and is currently under evaluation in a phase III clinical trial in Europe (CAPTURE) ^14^. This technology is viewed as promising but not as far along in development as INTERCEPT or Mirasol.

## Benefits

First and foremost, the most significant benefit of PRPs is the multi-log inactivation of susceptible blood-borne pathogens. While the supplementation of primary culture testing with other strategies like secondary culture or point of release testing can further mitigate the risk of bacterial contamination, PRPs have the added advantage of protecting the platelet supply from viruses and other nonbacterial agents, including new and emerging infectious agents. When new and emerging agents arise, transfusion recipients are even more vulnerable to TTIs during the lengthy period of time that it typically takes for the risk to be recognized and for protective measures to be implemented. PRT takes a proactive approach against pathogens that are not yet recognized as posing a risk for TTIs ^15^. Furthermore, PRT may avoid the need to develop and implement expensive and time-consuming algorithms for screening and testing for new infectious agents and provides an alternative approach to address certain low-prevalence or ‘tolerable’ risks such as Zika virus ^16^. If all types of blood products can be successfully treated with PRT, it may even be possible to envision a future where the residual risk of TTI is so low that infectious disease marker (IDM) testing could be made less stringent.

Transfusion-associated graft-versus-host disease (TA-GVHD) is a serious complication of transfusion caused by the viable donor lymphocytes present in the blood product. When transfusion recipients are severely immunocompromised or when donor and recipient HLA types are similar, irradiated blood products are required to reduce the risk of developing TA-GVHD. A second benefit of PRT is the inactivation of donor lymphocytes, which effectively protects transfusion recipients from developing TA-GVHD; PRPs are approved for this indication ^8^.

Currently, in most countries, including the US, PRPs can be stored for only 5 days. However, the FDA does allow 7-day storage of conventional platelets (CPs) when they are collected in specially approved containers and subjected to specific secondary culture or secondary point of release testing. Therefore, in the future, PRPs could be approved for 7-day storage, which would provide another strategy to help reduce platelet outdating, improve platelet availability, and ease platelet inventory management.

## Subjects of debate

Despite the benefits previously discussed, there are several topics of debate worthy of discussion. First, PRT offers incredible enhancement towards microbial safety. However, pyrogenic cell wall components, endotoxins, biofilm-positive isolates, spore-forming bacteria and non-enveloped viruses, and prions, while exceedingly rare, remain infectious risks transmissible through transfusion even after treatment with PRT ^17– 21^.

Second, there are concerns regarding the clinical efficacy of PRPs, in part because of initial data reporting decreased *in vitro* survival of PRPs compared to CPs ^22, 23^. The euroSPRITE, controlled, randomized, double-blinded study demonstrated that both 1-hour (hr) post-transfusion platelet count increment (PPCI) and 24-hr PPCI did not differ significantly between INTERCEPT PRPs and CPs ^22^. Subsequently, the randomized, controlled, SPRINT trial reported that while the incidence of World Health Organization (WHO) grade 2, 3, and 4 bleeding was equivalent, 1-hr corrected count increment (CCI) and mean days to next transfusion were decreased for INTERCEPT PRPs when compared to CPs ^23^. A meta-analysis of 12 clinical trials that assessed hematology oncology patients found moderate-quality evidence that transfusion of PRPs does not affect the risk of clinically significant or severe bleeding and high-quality evidence that PRP transfusions increase platelet transfusion requirement ^24^. Subsequent to the publication of this meta-analysis, three clinical studies were conducted to answer the question of whether PRPs are noninferior to CPs with regard to prevention of WHO grade 2 or higher bleeding in thrombocytopenic patients with hematologic malignancies ^25– 27^. The first study was not able to establish noninferiority of PRPs owing to low statistical power ^25^. The EFFIPSP study concluded that INTERCEPT PRPs are noninferior to platelets in additive solution, but noninferiority was not achieved when comparing INTERCEPT PRPs with platelets suspended in plasma ^26^. The PREPAReS trial evaluated the performance of Mirasol PRPs compared to CPs in plasma prepared from whole blood by the buffy coat method. This study demonstrated noninferiority in the intention to treat analysis, but noninferiority was not established in the per-protocol analysis ^27^. Unfortunately, noninferiority studies cannot demonstrate superiority of PRPs over CPs, or vice versa ^28^. In addition, the majority of bleeding events studied in these noninferiority studies were WHO grade 2, with insufficient statistical power to show a difference with WHO grade 3 or grade 4 bleeding because of the infrequency of these more severe bleeding outcomes.

Most studies evaluating the clinical efficacy of PRPs have been performed in adult hematology oncology patients. The therapeutic efficacy of PRPs in other patient populations such as pediatric patients or patients undergoing massive transfusion (MT) in the setting of trauma, organ transplantation, or surgery has not been adequately studied, but there are no a priori reasons to believe that PRPs would not provide similar clinical efficacy in these populations.

Rotational thromboelastometry (ROTEM) *in vitro* simulations of transfusion support in trauma using PRT-treated products versus untreated plasma and platelets showed no significant difference in ROTEM parameters when 30% of products were PRT treated but showed decreased hemostatic activity when 50% or greater PRT-treated plasma and platelets were used ^29^. A retrospective cohort analysis of 306 patients who had MTs in the setting of trauma, liver transplant, and cardiac and vascular surgery found that the introduction of INTERCEPT PRPs did not adversely affect clinical outcomes measured by blood product usage, in-hospital mortality, and length of stay ^30^.

Clinical efficacy data in the pediatric population exist but are limited. A retrospective study found similar utilization patterns for red blood cells but noted increased platelet transfusions in children aged 1–18 following the transfusion of INTERCEPT PRPs ^31^. Another retrospective evaluation of a total of 137 pediatric patients reported lower post-transfusion platelet counts, as well as lower 1-hr, 4-hr, 18-hr, and 24-hr CCIs in the Mirasol PRP group compared to the CP group, but the incidence of bleeding events did not differ ^32^. An observational study found a significant increase in platelet transfusion in Mirasol-treated platelet transfusions (458) compared to CP transfusions (176) in neonates ^33^. Additional studies are required to assess the efficacy of PRPs in pediatric patients as well as in those undergoing massive hemorrhage ^24^

Third, as with any new therapy, the safety profile of the product, namely the risk of transfusion-related adverse events (TRAEs), must be scrutinized. There is potential for UV exposure and/or residual photo-active compounds and byproducts to have short-term or long-term adverse effects ^34– 36^. For the INTERCEPT technology, a specific concern is related to the possibility of residual psoralen causing skin rashes in neonates who required phototherapy for hyperbilirubinemia ^37^. However, post-market surveillance conducted at an academic tertiary care medical center reported no episode of new rash in 11 neonatal intensive care patients undergoing phototherapy and transfused INTERCEPT PRP ^31^.

Another concern in patients of all ages is the risk of acute respiratory distress syndrome (ARDS). In the US, the INTERCEPT product is labeled with the precaution to monitor patients for signs and symptoms of ARDS, as patients receiving PRPs in the SPRINT trial had a higher (1.6% versus 0%) rate of developing ARDS ^23^. The PIPER trial is specifically designed to capture ARDS and clinically serious pulmonary adverse events ^38^. Of note, the Swiss hemovigilance data have reported a favorable safety profile for INTERCEPT platelets ^11^. Nonetheless, safety remains a concern in the vulnerable pediatric population because children are generally believed to be at an increased risk for TRAEs, and findings from studies in adult patients cannot be readily extrapolated to pediatric patients ^39^. A large Austrian regional medical center that provides transfusion support to hematology oncology and cardiac surgery as well as pediatric and neonatal patient populations reported only mild TRAEs that were not significantly different between CPs and INTECEPT platelets ^40^. Over a 5-year period, 379 children and 1,980 adults were transfused Mirasol platelets in the Balearic Islands of Spain and only mild TRAEs were observed ^33^. While the PIPER study will likely be informative, additional studies will still be required to determine if there are any long-term toxic effects of PRPs.

Another subject for deliberation is the risk of HLA alloimmunization. This topic was recently reviewed by Stolla ^41^. Studies in mouse and dog models have reported reduced alloimmunization rates after the transfusion of platelets treated with riboflavin and UV ^42, 43^. In contrast, the PREPAReS trial found a significantly increased rate of alloimmunization to HLA class I antigens ^44^. For INTERCEPT, the SPRINT trial found no significant difference in HLA antibody formation ^23^. These inconsistencies between animal studies and clinical trials and the variability noted in different trials may be due to the relative rare incidence of alloimmunization making it difficult to assess the increased risk of alloimmunization. Additional data from large clinical trials, such as data collected during the EFFIPAP trial and data currently being collected in the MIPLATE trial, may provide much-needed additional insight.

Lastly, the financial impact of implementing PRPs must be addressed. While initial data from the euroSPRITE and SPRINT trials raised the possibility that the use of PRPs may increase platelet utilization, several post-market studies have not documented increased platelet utilization ^45, 46^ nor have plasma and red cell utilization differed significantly ^40^. It is uncertain if an overall increase in blood products transfused would contribute to an increased cost associated with the implementation of PRPs. It is known that the per unit acquisition cost of PRPs is more than that of CPs ^47^. Financial considerations have stalled the widespread implementation of PRPs in the US ^15^ but non-financial considerations may also impede adoption in low- and middle-income countries ^48, 49^. In the future, the cost of PRP implementation could potentially be offset by approval for 7-day storage for PRPs ^50^ and possibly by reducing the need for developing and performing testing for new and emerging infectious agents that threaten the blood supply. Switzerland has taken this stance and deemed that the costs of PRPs are acceptable for improving transfusion safety ^11^. A recently published budget impact analysis (BIA) that modeled a mid-sized US hospital predicted a minimal cost increase for PRPs compared to CPs ^51^. However, an Italian BIA study concluded that further studies were needed to establish the cost-to-benefit ratio of PRPs ^52^. The debate regarding the financial and economic impact of PRPs is extremely difficult to resolve because it is not only challenging to determine cost but also impossible and, more importantly, inappropriate to extrapolate these findings from one country to another because of vastly different blood collection, healthcare, and reimbursement systems.

## What does the future hold?

Assuming that therapeutic efficacy is maintained and concerns over financial cost can be addressed, a blood supply that is completely treated by PRT capable of meaningfully reducing the risk of TTIs would provide a safeguard against currently known, new, and emerging infectious agents. This could possibly reduce donor screening and IDM testing and may even expand the donor pool and increase the availability of platelets.

With currently available PRT, IDM testing must continue, as variable levels of inactivation have been observed for bacteria and viruses, and none for prions ^53– 55^. Even with the multi-log inactivation of bacteria, on 14 June 2019 the Center for Disease Control and Prevention in the Morbidity and Mortality Weekly Report described a patient who had a STR after transfusion of PRPs postulated to be caused by post-treatment bacterial contamination ^56^. Currently, PRPs require not only IDM testing but also diligent monitoring and reporting of suspected STRs. However, the PRT available today is first generation. The use of other light sources with or without photo-active compounds is likely to emerge in the future; for example, the use of visible blue light to inactivate porphyrin molecules found in pathogens is a possible novel approach for manufacturing PRPs ^15^. As the storage of platelets at room temperature is the prime driver of the increased risk of bacterial contamination, there is also renewed interest in approaches that preserve platelet function during refrigerated storage or when platelets are cryopreserved ^57– 59^. It is critical that we re-evaluate the cost, risks, and benefits of new technologies as they become available, review the latest research and available evidence, and adjust our paradigm accordingly.

## Unresolved questions

Understanding the current literature on PRPs and recognizing the gaps in our knowledge help to identify areas for further investigation. Additional research to address the following questions is highly desirable.

1. Is it possible for new PRT to eliminate the risk posed by biofilm-positive isolates, pyrogenic cell wall components, endotoxins, bacterial spores, non-enveloped viruses, and prions?
2. Are PRPs noninferior to CPs in pediatric patients and actively bleeding patients requiring MT?
3. Do PRPs increase the risk of alloimmunization?
4. What is the long-term safety profile of PRPs, especially when given to neonates and children, patient populations with significant lifespans post-transfusion?
5. How does PRP implementation impact healthcare cost?

The answers to these questions are important from the perspective of patient care, patient safety, and health economics and will therefore direct the pace and breadth of PRT adoption by the international blood banking community.
